# Predictors of Ocrelizumab Effectiveness in Patients with Multiple Sclerosis

**DOI:** 10.1007/s13311-021-01104-8

**Published:** 2021-09-22

**Authors:** Maria Cellerino, Giacomo Boffa, Caterina Lapucci, Francesco Tazza, Elvira Sbragia, Elisabetta Mancuso, Nicolò Bruschi, Simona Minguzzi, Federico Ivaldi, Ilaria Poirè, Alice Laroni, Gianluigi Mancardi, Elisabetta Capello, Antonio Uccelli, Giovanni Novi, Matilde Inglese

**Affiliations:** 1 Department of Neuroscience, Rehabilitation, Ophthalmology, Genetics, Maternal and Child Health (DiNOGMI), University of Genoa, Largo Paolo Daneo 3, 16100 Genoa, Italy; 2 Laboratory of Experimental Neurosciences, IRCCS Ospedale Policlinico San Martino, Genoa, Italy; 3 Ospedale Policlinico San Martino-IRCCS, Genoa, Italy; 4 Scientific Clinical Institutes Maugeri IRCCS, Pavia, Italy; 5 Departments of Neurology, Radiology and Neuroscience, Icahn School of Medicine at Mount Sinai, NY New York, USA

**Keywords:** Multiple sclerosis, Ocrelizumab, Highly active multiple sclerosis, Advanced multiple sclerosis, CD8

## Abstract

Data regarding effectiveness and safety of ocrelizumab in the post-marking setting are lacking. The aim of our study was to provide effectiveness and safety data of ocrelizumab treatment in patients with relapsing–remitting (RR-) and progressive multiple sclerosis (PMS) and to evaluate clinical and immunological predictors of early treatment response. In this single-center prospective observational study, we investigated effectiveness outcomes (time-to-confirmed disability worsening, time-to-first relapse, time-to-first evidence of MRI activity and time-to-first evidence of disease activity), clinical and immunological predictors of early treatment response, and incidence of adverse events (AEs). One hundred and fifty-three subjects were included (93 RRMS; 84 females). Median follow-up was 1.9 (1.3–2.7). At 2-year follow-up (FU), disability worsening-free survival were 90.5%, 64.7%, and 68.8% for RRMS, primary-progressive MS (PPMS), and secondary-progressive MS (SPMS) patients, respectively. At 2-year FU, 67.1%, 72.7%, and 81.3% of patients with RRMS, PPMS, and SPMS were free of MRI activity, with NEDA-3 percentages of 62.1%, 54.6%, and 55.1%, respectively. Lower baseline EDSS was independently associated with a reduced risk of disability worsening (HR(95%CI) = 1.45(1.05–2.00), *p* = 0.024) and previous treatment exposure was independently associated with increased probability of radiological activity (HR = 2.53(1.05–6.10), *p* = 0.039). At 6-month FU, CD8 + cell decrease was less pronounced in patients with inflammatory activity (*p* = 0.022). Six patients (3.9%) discontinued ocrelizumab due to severe AEs. Our findings suggest that ocrelizumab is an effective treatment in real-world patients with RRMS and PMS, with a manageable safety profile. Better outcomes were observed in treatment-naïve patients and in patients with a low baseline disability level. Depletion of CD8 + cells could underlie early therapeutic effects of ocrelizumab.

**Supplementary Information:**

The online version contains supplementary material available at 10.1007/s13311-021-01104-8.

## Background

Despite an increasing number of approved disease-modifying treatments (DMT) to reduce inflammatory activity, multiple sclerosis (MS) remains a leading cause of neurological disability in young adults [1]. Ocrelizumab is a B-cell depleting drug recently approved for the treatment of MS. Phase III clinical trials have shown significant benefits of ocrelizumab use in terms of clinical and MRI outcomes in relapsing–remitting (RR) and primary-progressive (PP) MS, along with a manageable safety profile [2, 3]. Open-label extension phases of pivotal trials confirmed that earlier and continuous treatment with ocrelizumab can provide sustained disease control in patients with RRMS [4] and PPMS [5]. Subgroup and post hoc analyses of efficacy endpoints from the pooled OPERA I and OPERA II populations have been reported [6–8], showing that the treatment effect of ocrelizumab versus IFN β-1a was maintained across most of the subgroups and strata of interest. Moreover, a recently published post hoc exploratory analysis provided effectiveness data of ocrelizumab in a subgroup of patients with advanced MS [9].

However, available observational studies regarding its use in the post-marketing setting are still sparse [10–12] and characterized by several limitations, including the retrospective nature, limited numbers of patients enrolled, short follow-up duration, lack of MRI, and/or immunological data. Complementing the results of phase III clinical trials with observational data referring to an “unselected cohort” is critical in order to achieve information that could be transferred to real-life scenarios. Post-marketing studies exploring the onset of efficacy after treatment commencement and the clinical and immunological variables associated with outcomes are indeed warranted, especially for patients with highly active forms of MS and advanced progressive MS.

Moreover, due to the inclusion of a highly selected population, adverse events might be also underestimated in pivotal trials [13, 14]. Since the positioning of ocrelizumab is still not well established within the therapeutic algorithm for MS, it is crucial to provide clinicians with relevant information on prognostic and predictive factors that can help assess the benefit-risk ratio.

Therefore, the aim of our study was to investigate in a real-world setting the effectiveness and safety of treatment with ocrelizumab in patients with RR and progressive MS (PMS) and to determine potential clinical or immunological predictors of treatment response.

## Methods

### Study Design, Standard Protocol Approvals, and Patient Consent

This is an observational prospective single-center cohort study conducted at the MS Center of the University of Genoa, Policlinico San Martino, evaluating effectiveness and safety of ocrelizumab therapy for the treatment of RRMS and PMS. All participants provided consent to use their medical history for publication. Ocrelizumab was prescribed and administered by the treating physician to relapsing forms of MS and early PPMS according to regulatory policies. From March to June 2020, due to coronavirus disease 2019 (COVID19) pandemic, ocrelizumab was administered following a tailored approach evaluating the profile risk of each patient, according to international indications [15, 16]. All patients were clinically evaluated every 3 months for assessment of effectiveness and safety. One-hundred forty-five patients (95%) underwent baseline and follow-up MRI scans at our institution with a standardized 3-T MRI protocol (Siemens PRISMA). A subset of patients (*n* = 73) underwent serial blood samples at our institution every 6 months, before ocrelizumab infusion, for lymphocyte profiling and immunoglobulin concentration.

### Study Endpoints

The primary objective of our study was to determine time-to-first relapse, time-to-confirmed disability worsening, time-to-first evidence of MRI activity, and time-to-first evidence of disease activity (according to the NEDA-3 definition) in real-life patients treated with ocrelizumab. Secondary outcomes were to (i) assess efficacy outcomes in patients with recent high disease activity and those with a relatively high disability at ocrelizumab commencement, (ii) assess clinical and immunological predictors of early inflammatory activity based on flow-cytometry immune subsets characterization during ocrelizumab therapy, and (iii) assess safety of treatment. Baseline brain MRI (acquired within 3 months before ocrelizumab start) was the pre-treatment reference scan for assessment of treatment failure. As exploratory analyses, re-baseline of MRI activity was performed 100 and 180 days after ocrelizumab start. Recent highly active MS was defined, based on the criteria proposed by Rush et al. [17], as the presence of MRI activity and at least one relapse in the year before ocrelizumab start in patients with accelerated accrual of disability (EDSS ≥ 4.0). In line with post hoc analyses of OPERA and ORATORIO studies [9], we separately explored efficacy outcomes in patients with baseline EDSS score ≥ 4 at ocrelizumab start. Early inflammatory activity was defined as the occurrence of MRI inflammatory activity or a relapse within the first 12 months of treatment. All the analyses were performed on the global cohort of patients and, as sensitivity analyses, in the cohort of patients with RRMS and progressive MS separately.

### Statistical Analyses

Descriptive results were reported as mean with standard deviation (SD) or median with interquartile range (IQR). The probability of disability worsening-free survival, relapse-free survival, MRI activity-free survival, and NEDA-3 status was calculated with the Kaplan–Meier estimator. Univariate and multivariate analyses assessing the association of demographic- and disease-related characteristics with survival endpoints were performed using Cox proportional hazards regression analysis models. Variables significantly associated with each outcome event on univariate analysis were included as covariates in the multivariate model. Differences in lymphocyte subpopulations at different timepoints were assessed with analysis of covariance, adjusting for age, sex, MS phenotype, and last DMT before ocrelizumab initiation. Correction was made for multiple comparisons (Bonferroni-*p* = 0.0028). Univariate and multivariate binary logistic regression analyses were used to explore the predictive role of clinical and immunological variables in terms of early inflammatory activity. A time*early treatment response group interaction was included into a linear mixed model with random intercept and random slope to test differences on lymphocyte subset values time trend between patients with and without early inflammatory activity. A two-sided *p* < 0.05 was used for statistical significance. SPSS 23 (IBM; version 23.0) and R software (version 4.0.3) were used for computation.

## Results

### Study Population

One hundred and fifty-three consecutive MS patients (93 RRMS, 43 PPMS and 17 relapsing, secondary-progressive (SP) MS) initiated treatment with ocrelizumab at the MS Center of the University of Genoa, Policlinico IRCCS San Martino, from July 2017 to July 2020. Demographic- and disease-related characteristics at ocrelizumab start are reported in Table 1.

**Table 1:** Demographic and clinical characteristics

	**Total cohort** (*n* = 153)	**RRMS** (*n* = 93)	**Progressive-MS**		
			**PPMS** (*n* = 43)	**SPMS** (*n* = 17)	**Total PMS** (*n* = 60)
Females, *n* (%)	84 (54.9)	60 (64.5)	20 (46.5)	4 (23.5)	24 (40.0)
Age, mean (SD), y	41.9 (11.4)	36.9 (10.2)	49.2 (8.6)	50.6 (8.2)	49.6 (8.4)
Disease duration, mean (SD), y	10.3 (9.9)	9.3 (9.2)	8.4 (6.5)	20.8 (14.7)	11.9 (11.0)
EDSS, median (IQR)	3.5 (2–5.5)	2 (2–3.5)	5.5 (3.5–6.5)	6 (5–6.5)	6 (3.5–6.5)
Number of relapses in previous 12 months, mean (SD)	0.5 (0.7)	0.8 (0.7)	-	0.2 (0.4)	0.1 (0.2)
MRI activity at ocrelizumab start, *n* (%)	91 (59.5%)	75 (81.5)	9 (21.4)	7 (41.2)	16 (27.1)
Number of previous treatment, median (IQR)	1 (0–2)	1 (0–3)	1 (0–1)	1 (1–3)	1 (0–2)
Naïve patients, *n* (%)	46 (30.1)	25 (26.9)	18 (41.9)	3 (17.6)	21 (35.0)
Previous exposure to high efficacy DMT, *n* (%)	58 (54.2)	44 (64.7)	7 (28.0)	7 (50.0)	14 (35.9)
Last DMT, *n* (%)					
Interferon	12 (7.8)	7 (7.5)	4 (9.3)	1 (5.9)	5 (8.3)
Glatiramer acetate	10 (6.5)	4 (4.3)	3 (7.0)	3 (17.6)	6 (10.0)
Fingolimod	26 (17)	24 (25.8)	1 (2.3)	1 (5.9)	2 (3.3)
Dimethyl fumarate	13 (8.5)	6 (6.5)	5 (11.6)	2 (11.8)	7 (11.7)
Teriflunomide	4 (2.6)	4 (4.3)	0 (0)	0 (0)	0 (0)
Natalizumab	9 (5.9)	6 (6.5)	0 (0)	3 (17.6)	3 (5.0)
Alemtuzumab	11 (7.2)	10 (10.8)	0 (0)	1 (5.9)	1 (1.7)
Cladribine	2 (1.3)	2 (2.2)	0 (0)	0 (0)	0 (0)
Other	20 (13.1)	5 (5.4)	12 (27.9)	3 (17.6)	15 (25)
Time from DMT discontinuation to ocrelizumab start, median (IQR), d	68 (30–501)	60 (25–112)	335 (81–1157)	859 (132–2158)	452 (99–1228)
Highly active MS, *n* (%)	17 (11.1)	14 (15.1)	-	3 (17.6)	3 (5.0)
Advanced MS, *n* (%)	66 (43.1)	22 (23.7)	30 (69.8)	14 (82.4)	44 (73.3)
Follow-up, median (IQR), y	1.9 (1.3–2.7)	1.6 (1.11–2.03)	2.5 (2.0–3.0)	2.0 (1.2–2.7)	2.3 (1.8–2.99)

*RRMS* relapsing–remitting multiple sclerosis, *PPMS* primary-progressive multiple sclerosis, *SPMS* secondary progressive multiple sclerosis, *PMS* progressive multiple sclerosis, *n* number, *SD* standard deviation, *IQR* interquartile range, *EDSS* expanded disability status scale, *DMT* disease-modifying therapy

### Primary Outcome

**Fig. 1:**
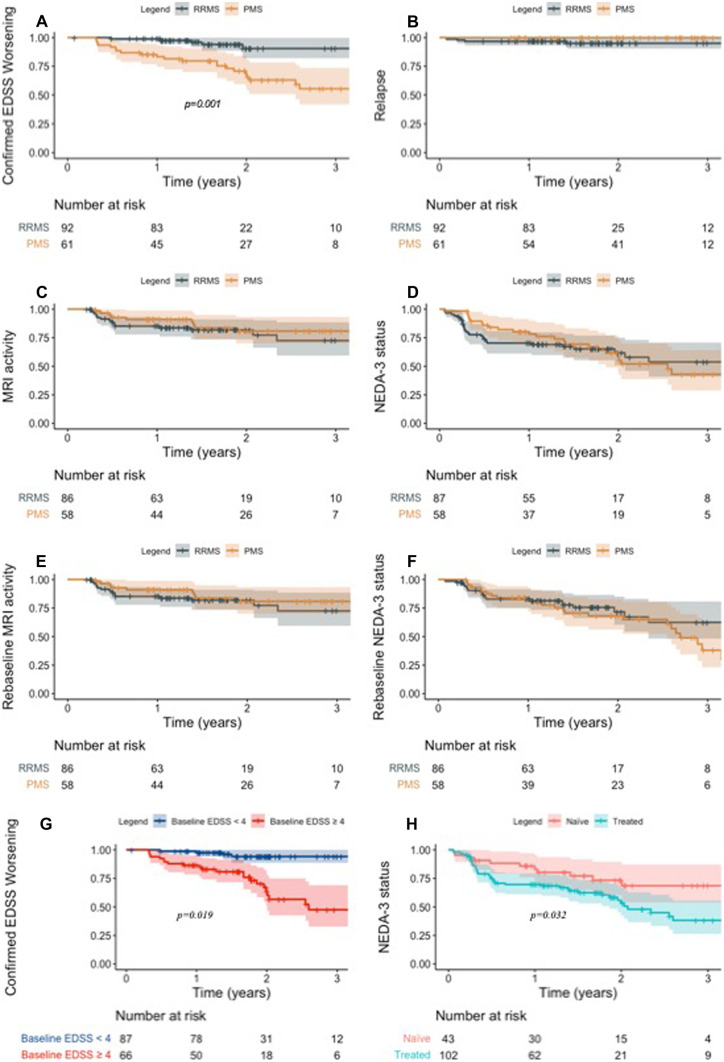
NEDA-3 status and individual components during ocrelizumab therapy in relapsing–remitting and progressive MS. Specifically, panel (**A**) shows the percentages of RRMS and PMS patients free of disability worsening throughout follow-up; panel (**B**) shows occurrence of relapses in RRMS and PMS throughout follow-up; panel (**C)** shows RRMS and PMS patients free of MRI activity throughout follow-up; panel (**D**) NEDA-3 percentages in RRMS and PMS patients throughout follow-up; panel (**E**) shows RRMS and PMS patients free of MRI activity throughout follow-up after a 100-day re-baseline of MRI activity; panel (**F**) shows NEDA-3 percentages in RRMS and PMS patients throughout follow-up after a 100-day re-baseline of MRI activity; panel (**G**) shows percentages of subjects free of disability worsening throughout follow-up in sub-groups of patients with baseline EDSS<4 and those with baseline EDSS ≥ 4 at ocrelizumab start; panel (**H**) shows NEDA-3 percentages in treatment-naive and previously treated patients throughout follow-up. RRMS, relapsing–remitting multiple sclerosis; PMS, progressive multiple sclerosis; EDSS, expanded disability status scale; NEDA-3, no evidence of disease activity

Figure 1 (panel A–F) reports the results of the primary outcome. At 2-year FU, 90.5% of patients with RRMS, 64.7% of patients with PPMS, and 68.8% of patients with SPMS were free of disability worsening. Table 2 reports the results of the univariate and multivariate analyses of clinical factors influencing the outcomes. Lower EDSS, RRMS phenotype, shorter disease duration, younger age, higher ARR, and presence of inflammatory signs at baseline MRI were associated with reduced risk of disability worsening. At multivariate analyses, lower EDSS was independently associated with a better clinical outcome (HR 1.45 (1.05–2.00), *p* = 0.023). Two out of five patients with RRMS who experienced disability worsening had highly active MS and 5/5 experienced progression independent of relapse activity.

**Table 2:** Univariate and multivariate analyses of factors associated with outcomes

	**Disability worsening**		**MRI-inflammatory activity**		**NEDA-3 status**	
	**HR (95% CI)**	***p* value**	**HR (95% CI)**	***p* value**	**HR (95% CI)**	***p* value**
**Total cohort** (*n* = 153)						
MS phenotype, RRMS/PMS	5.69 (2.13–15.2)	0.001^#^	0.47 (0.23–0.98)	0.045°	0.98 (0.57–1.67)	0.931
Age	1.08 (1.04–1.12)	< 0.0001^#^	0.99 (0.96–1.05)	0.357	1.01 (0.99–1.04)	0.218
Sex, female/male	0.75 (0.35–1.62)	0.461	0.49 (0.24–1.00)	0.052°	1.28 (0.75–2.18)	0.372
Disease duration	1.03 (1.00–1.07)	0.036^#^	0.99 (0.95–1.02)	0.431	1.00 (0.97–1.03)	0.960
EDSS	1.74 (1.37–2.22)	< 0.0001^#^	0.98 (0.83–1.16)	0.847	1.14 (0.99–1.30)	0.060
Previous treatment, naïve/treated	1.68 (0.67–4.19)	0.268	0.42 (0.17.1.01)	0.052°	0.48 (0.25–0.94)	0.032
Active baseline MRI scan, yes/no	0.31 (0.13–0.75)	0.009^#^	2.65 (1.24–5.64)	0.012°	1.15 (0.67–1.97)	0.621
**RRMS** (*n* = 93)						
Age	1.08 (0.99–1.18)	0.086	1.01 (0.97–1.05)	0.617	1.02 (0.99–1.06)	0.223
Sex, female/male	7.31 (0.82–65.4)	0.075	1.44 (0.61–3.43)	0.408	0.85 (0.41–1.75)	0.662
Disease duration	1.08 (0.99–1.17)	0.063	0.97 (0.92–1.02)	0.268	0.98 (0.94–1.02)	0.374
EDSS	1.38 (0.84–2.28)	0.203	1.20 (0.94–1.55)	0.148	1.20 (0.95–1.50)	0.121
Previous treatment, naïve/treated	37.5 (0.01–112)	0.375	2.46 (0.84–7.19)	0.100	0.41 (0.15–1.08)	0.075
Active baseline MRI scan, yes/no	0.90 (0.10–8.15)	0.927	1.83 (0.55–6.10)	0.325	1.53 (0.53–4.41)	0.426
**Progressive MS** (*n* = 60)						
Age	1.04 (0.99–1.10)	0.149	0.99 (0.93–1.07)	0.861	1.02 (0.97–1.06)	0.479
Sex, female/male	1.90 (0.81–4.50)	0.141	2.44 (0.69–8.64)	0.168	2.22 (0.99–4.98)	0.054
Disease duration	1.01 (0.98–1.05)	0.519	1.01 (0.97–1.06)	0.569	1.01 (0.98–1.05)	0.428
EDSS	1.66 (1.16–2.37)	0.005	1.11 (0.75–1.66)	0.589	1.30 (0.98–1.73)	0.069
Previous treatment, naïve/treated	1.39 (0.54–3.59)	0.494	1.95 (0.41–9.19)	0.399	1.48 (0.59–3.75)	0.406
Active baseline MRI scan, yes/no	0.68 (0.20–2.33)	0.535	3.31 (0.92–11.9)	0.067	0.99 (0.34–2.93)	0.992

*n* number, *HR* hazard ratio, *95% CI* confidence intervals, *RRMS* relapsing–remitting multiple sclerosis, *PMS* progressive multiple sclerosis, *EDSS* expanded disability status scale, *NEDA-3* no evidence of disease activity

^#^Multivariate analysis: Chi-square = 24, *p* < 0.0001, EDSS = [HR (95%CI) = 1.45 (1.05–2.00), *p* = 0.024]

°Multivariate analysis; Chi-square = 14, *p* = 0.006, previous treatment, naive/treated = [HR (95%CI) = 2.53 (1.05–6.10), *p* = 0.039]

A total of 95.1% patients with RRMS were free of relapses at 2-year FU. Four patients had a single relapse at + 27, + 72, + 103, and + 520 days, respectively. In the RRMS cohort, pre-treatment annualized relapse rate (ARR) was 0.78 (0.70), 1-year FU ARR was 0.04 (0.18), and 2-year FU  ARR was 0.04 (0.21).

At 2-year FU, 67.1% RRMS patients, 81.3% PPMS patients, and 72.7% SPMS patients were free of MRI evidence of disease activity. After a 100-day re-baseline of MRI activity, percentages of patients without MRI activity increased to 82.1%, 83.8%, and 72.7% for RRMS, PPMS, and SPMS patients respectively. After a 180-day re-baseline, percentages increased to 92.1%, 88.4%, and 83.1%. Male sex, RRMS phenotype, previous exposure to DMT, and baseline active MRI were associated with an increased risk of MRI activity during ocrelizumab therapy. At multivariate analysis, patients previously treated with a DMT had an increased risk of MRI activity (HR (95%CI) = 2.53 (1.05–6.10), *p* = 0.039). Among 21 patients who experienced MRI inflammatory activity within 180 days, only 1 patient had further MRI activity at + 520 days.

At 2-year FU, NEDA-3 percentages were 62.1%, 54.6%, and 55.1% for RRMS, PPMS, and SPMS respectively. After a 100-day re-baseline of MRI activity, NEDA-3 percentages increased to 71.9%, 71.4%, and 57.9% respectively. NEDA-3 rates after a 180-day re-baseline were 77.1%, 71.9%, and 67.3% for RRMS, PPMS, and SPMS respectively. Naïve patients had a higher probability of achieving NEDA-3 status (HR 0.48 (0.25–0.94), *p* = 0.032) (Fig. 1, panel H).

### Patients with Baseline EDSS ≥ 4 and with Highly Active MS

Patients with baseline EDSS ≥ 4 at ocrelizumab start had a higher risk of disability worsening (disability worsening-free survival 60.1% vs 94.1%, HR = 8.16 (2.81–23.7), *p* < 0.0001) and lower NEDA-3 rates (43.4% vs 70.1%, HR = 1.90 (1.11–3.24), *p* = 0.019) compared to patients with baseline EDSS < 4 (Fig. 1, panel G). Patients with highly active MS had a higher risk of persistent MRI inflammatory activity (MRI-free survival 58.3% vs 75.5%, HR = 0.35 (0.15–0.82), *p* = 0.015) and tended to have lower NEDA-3 rates (NEDA-3 percentage of 38.9% vs 60.1, HR = 0.48 (0.22–1.02), *p* = 0.056) compared to patients without the same baseline characteristics.

### Predictors of Early Inflammatory Activity

Out of 145 patients, 30 (20.7%) had early inflammatory activity in the first year of treatment. The RRMS phenotype (OR 3.19 (1.21–8.39), *p* = 0.019) and MRI activity at baseline (OR 0.21 (0.07–0.59), *p* = 0.003) were associated with an increased risk of early inflammatory activity. Leukocyte, total lymphocyte, CD4 + , CD8 + , and CD19 + cell counts during the first year of ocrelizumab therapy are reported in Table 3. While no significant differences in cell count were present at baseline, at 6-month FU, patients with early inflammatory activity had higher total lymphocytes, CD3 + , CD4 + , and CD8 + cell counts compared with stable patients. Only difference in CD8 + cell count survived Bonferroni correction for multiple comparisons. Using linear mixed model, CD8 + cell decrease at 6-month FU was less pronounced in patients with early inflammatory activity (*p* = 0.022), while no significant differences were noted in the dynamics of CD4 + and CD19 + counts. When including CD4 + , CD8 + , and CD19 + cell counts to MS phenotype and MRI baseline activity in a logistic regression model predicting early inflammatory activity, CD8 + count was the only independent variable associated with outcome (OR 1.005 (1.001–1.009), *p* = 0.019).

**Table 3:** Lymphocyte subsets during the first year of treatment

	**Total cohort**	**Treated**	**Treatment-naive**	**Treated vs naive** ***p* value^**	**With early inflammatory activity**	**Without early inflammatory activity**	**With vs without early inflammatory activity** ***p* value°**
**Baseline**							
Available, *n*	73	55	18		17	56	
Leukocytes, N/mm^3^	6273 (2262)	6064 (2208)	6913 (2368)	0.254	6501 (3232)	6205 (1908)	0.522
Lymphocyte, N/mm^3^	1793 (771)	1680 (801)	2139 (557)	0.050	2030 (1188)	1721 (587)	0.088
CD3 + , N/mm^3^	1259 (602)	1158 (624)	1568 (404)	0.022*	1452 (879)	1201 (484)	0.085
CD4 + , N/mm^3^	807 (446)	742 (463)	1006 (323)	0.047*	922 (641)	772 (367)	0.172
CD8 + , N/mm^3^	439 (214)	411 (224)	526 (153)	0.088	515 (263)	416 (193)	0.064
CD19 + , N/mm^3^	291 (257)	289 (244)	299 (303)	0.960	334 (366)	279 (217)	0.407
**6 months**							
Available, *n*	73	55	18		17	56	
Leukocytes, N/mm^3^	5712 (1666)	5681 (1737)	5806 (1469)	0.870	5991 (1386)	5627 (1745)	0.209
Lymphocyte, N/mm^3^	1388 (547)	1306 (554)	1639 (451)	0.036*	1564 (666)	1335 (501)	0.049*
CD3 + , N/mm^3^	1122 (505)	1042 (508)	1367 (419)	0.027*	1327 (617)	1060 (454)	0.014*
CD4 + , N/mm^3^	714 (367)	652 (366)	903 (309)	0.016*	824 (446)	680 (338)	0.046*
CD8 + , N/mm^3^	369 (192)	352 (196)	421 (176)	0.305	484 (214)	334 (173)	0.001**
CD19 + , N/mm^3^	19 (32)	23 (36)	7 (11)	0.084	29 (46)	17 (27)	0.495
**12 months**							
Available, *n*	61	45	16		11	50	
Leukocytes, N/mm^3^	6306 (2704)	6296 (3055)	6334 (1314)	0.912	5432 (1499)	6532 (2926)	0.482
Lymphocyte, N/mm^3^	1488 (655)	1359 (669)	1868 (476)	0.022*	1513 (561)	1528 (661)	0.584
CD3 + , N/mm^3^	1204 (524)	1091 (496)	1525 (476)	0.012*	1233 (493)	1211 (533)	0.469
CD4 + , N/mm^3^	780 (356)	695 (328)	1020 (330)	0.003*	760 (320)	794 (365)	0.759
CD8 + , N/mm^3^	397 (224)	370 (228)	471 (199)	0.251	456 (314)	387 (201)	0.199
CD19 + , N/mm^3^	25 (70)	32 (81)	5 (7)	0.267	11 (14)	28 (78)	0.314

^Multivariate ANCOVA corrected for age, sex, MS phenotype

°Multivariate ANCOVA corrected for age, sex, MS phenotype, and last DMT before ocrelizumab initiation

^*^ Uncorrected *p* < 0.05

^**^Bonferroni *p* < 0.0028

### Safety

Adverse events and serious adverse events are detailed in Table 4. Seventy-four patients (48.4%) experienced at least one adverse event while on ocrelizumab therapy. Mean (SD) number of adverse events per patient was 1.8 (1.3). Infections accounted for the vast majority (89.2%) of total adverse events. Patients with PMS had a slightly increased risk of adverse events (OR = 0.52 (0.27–0.99), *p* = 0.049). No differences in the frequencies of total and infectious adverse events were noted between treatment-naïve patients and patients previously treated with other DMTs. Most patients (74.3%) experienced the first adverse event within the first year of treatment. In five patients (3.3%), a diagnosis of neoplasm was reported: specifically breast cancer (*n* = 2, at + 50 and + 1090 days from first ocrelizumab course), cervical cancer (*n* = 1, + 113 days), lymphoma (*n* = 1, + 770 days), and a myelodysplastic syndrome eventually evolved into acute myeloid leukemia (*n* = 1, + 613 days). Four patients developed COVID-19, with mild disease in 3 of them. One patient developed moderate-severe disease requiring hospitalization, without need for intensive care unit admission. All patients underwent complete recovery. At 6-month FU, 7 patients (9.6%) developed IgG hypogammaglobulinemia, 1 patient (1.4%) developed IgA hypogammaglobulinemia, and 12 (16.4%) developed IgM hypogammaglobulinemia. At 12-month FU, the percentages of patients with IgG, IgA, and IgM hypogammaglobulinemia increased to 6.8%, 2.7%, and 23.3%, respectively. At 18-month FU, 2/40 patients (5%) had IgG hypogammaglobulinemia, 3 patients (7.5%) had IgA hypogammaglobulinemia, and 9 patients (22.5%) had IgM hypogammaglobulinemia. At 24-month FU, the frequency of hypogammaglobulinemia was 11.1% (3/27) for IgG, 0% for IgA, and 29.6% [8] for IgM. No patient developed severe IgG hypogammaglobulinemia (< 200 mg/dL) [18].

**Table 4:** Safety

	**Total cohort**	**RRMS**	**PMS**
Any adverse events, *n* (%)	74 (48.4)	39 (41.9)	35 (58.3)
Adverse events leading to ocrelizumab discontinuation, *n* (%)	6 (3.9)	3 (2.0)	2 (1.3)
Adverse events leading to hospitalization, *n* (%)	4 (2.6)	2 (1.3)	2 (1.3)
Number of adverse events per subject, median (IQR)	1 (1–2)	1 (1–2)	1 (1–2)
Time from first ocrelizumab infusion, d	161 (54–351)	120 (54–303)	206 (53–395)
Serious infusion associated reactions, *n* (%)	0	0	0
Infectious adverse events, *n* (%)	66 (43.1)	35 (37.6)	31 (51.7)
Pneumonia	2	2	0
Upper respiratory tract infection	40	16	24
Lower urinary tract infection	8	6	2
HSV1 reactivation	12	8	4
VZV reactivation	2	2	0
COVID	4	3	1
Neoplasm, *n* (%)	5 (3.3)	3 (2.0)	2 (1.3)
Death, *n* (%)	0	0	0

*RRMS* relapsing–remitting multiple sclerosis, *PMS* progressive multiple sclerosis, *n* number, *IQR* interquartile range, *HSV1* herpes simplex virus type 1, *VZV* varicella zoster virus, *COVID* coronavirus 19 associated disease

## Discussion

We herein provide single-center effectiveness and safety data about ocrelizumab treatment in relapsing–remitting and progressive MS patients, prospectively followed in a real-world setting for a median follow-up of almost 2 years. Our cohort consisted of a heterogeneous group of patients with a large variety in terms of age, disease activity, phenotype, treatment history, and comorbidities, and included a relatively high number of patients with recent high disease activity and relatively high disability at treatment start.

In the RRMS population we observed that, at 2-year FU, 95.1% patients were free of relapses, 90.5% patients free of disability worsening, and 67.1% patients free of MRI activity, with an overall percentage of patients reaching NEDA-3 of 62.1%. The ARR decreased from 0.78 to 0.04 (at 1- and 2-year FU), which is in line with the relapse rate at 2-year FU reported in the OPERA I and II studies (0.16) [2]*.*

As per the progressive cohort, we observed a percentage of patients free of disability worsening at 2-year FU of 64.7% and 68.8% in PPMS and SPMS, respectively. ORATORIO study was the first clinical trial to demonstrate a benefit on disability progression in PPMS patients treated with ocrelizumab, showing a percentage of disability worsening-free patients of 67.1% (3); its open-label extension confirmed the persistent treatment-associated beneficial effect in terms of disability progression over a period of 5 years [5]. Our results show a sustained beneficial effect of ocrelizumab treatment in progressive patients over a period of 2 years in a real-world setting. Of note, although no phase III clinical trials specifically exploring the effect of ocrelizumab use in SPMS patients is available, our data might suggest that relapsing SPMS respond to ocrelizumab similarly to PPMS patients.

When analyzing potential predictors of treatment response, lower baseline EDSS was independently associated with a reduced risk of disability worsening, while previous DMT exposure was independently associated with an increased risk of MRI activity and NEDA-3 failure. The effect of EDSS in influencing the risk of disability worsening was particularly evident in the cohort of patients with PMS. In line with this finding, we observed that patients with baseline EDSS ≥ 4 had a higher risk of disability worsening and lower NEDA-3 rates at 2-year FU. As for patients with RRMS, we observed a tendency towards a reduced risk of NEDA-3 failure in treatment-naïve patients. Taken together, our results suggest that early commencement with ocrelizumab treatment should be considered in order to prevent persistent disease activity. These finding are in line with recent evidence demonstrating that early commencement of highly efficacy drugs is associated with reduced long-term disability and later conversion to SPMS [19–21].

Despite such encouraging results, we observed some evidence of persistent MRI activity in the first year of treatment, both in RRMS and PMS patients (MRI activity-free survival at 2 years of 67.1%, 81.3%, and 72.7% for RRMS, PPMS, and SPMS patients, respectively). The post hoc analysis pooling results of phase II and III trials suggested that ocrelizumab efficacy in terms of MRI outcome is evident as early as 4 weeks, and nearly complete by week 8 [22]. We performed a re-baseline of MRI activity at 100 and 180 days after ocrelizumab start and observed that percentages of MRI activity-free patients increased to 73–82% and to 83–92%, respectively. Accordingly, NEDA-3 percentages after 180 days of treatment increased to 77.1%, 71.9%, and 67.3% for RRMS, PPMS, and SPMS respectively. These results are in line with the post hoc analysis of the OPERA trial, in which the proportion of patients reaching NEDA-3 status was 72.2% after a 24-week re-baseline [23]. In RRMS patients, the presence of inflammatory activity at baseline MRI and a previous DMT exposure were associated with an increased risk of MRI activity during ocrelizumab treatment, with previous DMT exposure being the only independent variable associated with worse outcome at the multivariate analysis. Accordingly, a previous DMT exposure was associated with lower probabilities of achieving NEDA-3 status. These results are in line with those from an independent cohort of patients treated with ocrelizumab, in which lower baseline EDSS and higher previous relapse rate were associated with an increased risk of early inflammatory activity during ocrelizumab treatment [24].

We also observed significantly lower rates of MRI activity-free survival (58.3%) in the subgroup of patients with highly active MS, compared to the total cohort of patients. Since a rapid effect in controlling MRI activity is critical to minimize brain damage and prevent accumulation of disability in aggressive MS, these results suggest that in patients in whom immediate and complete disease control is warranted, DMTs with more rapid immune-ablative action should be considered.

It is generally believed that the impairment of the antigen-presenting capacity of B cells is one of the major mechanisms underlying the therapeutic efficacy of ocrelizumab. However, a subset of T cells, mainly CD8 + , also expresses CD20 and represents a highly activated cell population [25]. CD20 + T cells have been found to be increased in MS patients [26], representing almost 20% of all CD20 + expressing cells [27]. It has been recently showed that ocrelizumab reduces CD20 + T cells after 6 months of treatment [28], in line with similar findings found during treatment with rituximab [29]. We showed that patients with persistent early inflammatory activity during the first year of treatment had higher levels of CD8 + cells at 6-month FU as compared with stable patients. Although our evidence is limited, we cautiously speculate that the decrease in CD8 + cells is driven, at least partially, by the reduction of CD20 + T cells induced by ocrelizumab. Indeed, a transient reduction in CD8 + cells has been reported in phase III trials [2, 3], and in real-life patients [30], but to date no evidence exists on the possible therapeutic effect of this cellular subset reduction. Since these findings are based on immunophenotyping data collected in clinical practice, we could not validate them with double staining analyses and thus provide a mechanistic explanations for our observations. Larger studies exploring the effect of ocrelizumab treatment on CD20 + CD8 + cells and its possible association with clinical outcomes are needed in order to confirm our findings.

In line with phase II and III clinical trials and the few real-world studies available, our data showed an overall favorable safety profile of ocrelizumab in RRMS and PMS. Of note, we observed a slightly increased risk of AEs among real-life progressive patients, as reported by other groups [31]. Upper respiratory tract infections represented the most frequently observed AEs. Concerns have been raised regarding an increased risk of infectious events due to sustained B-cell depletion over time. In our population, most AEs occurred within the first year of treatment. We cannot exclude that the restriction due to COVID19 pandemics implemented in the past months reduced the risk of other respiratory tract infection, as observed with influenza virus [32]. The frequency of malignancies observed in our study was 3.3% (2.3% in ORATORIO, 0.7% and 0.2% in OPERA I and II, respectively). These results underline the importance of strictly following the regular standard cancer screening according to guidelines.

Our study has several limitations, including the lack of spine MRI, patients’ BMI, and a relatively short follow-up. In addition, flow cytometry immune subset characterization was available only for a subgroup of patients. Finally, pre-treatment brain MRI were acquired within 3 months of ocrelizumab start and not just before treatment commencement; thus, we cannot rule out whether part of the persistent MRI activity we observed during the first months of treatment occurred before treatment start.

## Conclusions

Our data point at ocrelizumab as an effective treatment option in patients with RRMS and PMS, especially for those patients who initiate ocrelizumab treatment in the early phase of the disease and for treatment-naïve patients. Depletion of CD8 + cells could account for early therapeutic effects of ocrelizumab.

## Supplementary Information

Below is the link to the electronic supplementary material.

Supplementary file1 (PDF 525 KB)

Supplementary file2 (PDF 559 KB)

Supplementary file3 (PDF 610 KB)

Supplementary file4 (PDF 508 KB)

Supplementary file5 (PDF 645 KB)

Supplementary file6 (PDF 576 KB)

Supplementary file7 (PDF 525 KB)

Supplementary file8 (PDF 122 KB)

Supplementary file9 (PDF 122 KB)

Supplementary file10 (PDF 551 KB)

Supplementary file11 (PDF 568 KB)

Supplementary file12 (PDF 627 KB)

Supplementary file13 (PDF 585 KB)

Supplementary file14 (PDF 542 KB)

Supplementary file15 (PDF 534 KB)

Supplementary file16 (PDF 122 KB)

## Data Availability

The data that support the findings of this study are available from the corresponding author upon reasonable request.

## References

[1] Reich DS, Lucchinetti CF, Calabresi PA. Multiple Sclerosis. Longo DL, editor. N Engl J Med [Internet]. 2018 Jan 11;378(2):169–80. Available 10.1056/NEJMra1401483 10.1056/NEJMra1401483 PMC6942519 29320652

[2] Hauser SL, Bar-Or A, Comi G, Giovannoni G, Hartung H-P, Hemmer B, et al. Ocrelizumab versus Interferon Beta-1a in Relapsing Multiple Sclerosis. N Engl J Med [Internet]. 2017 Jan 19;376(3):221–34. Available from: 10.1056/NEJMoa1601277 10.1056/NEJMoa1601277 28002679

[3] Montalban X, Hauser SL, Kappos L, Arnold DL, Bar-Or A, Comi G, et al. Ocrelizumab versus Placebo in Primary Progressive Multiple Sclerosis. N Engl J Med [Internet]. 2017 Jan 19;376(3):209–20. Available from: 10.1056/NEJMoa1606468 10.1056/NEJMoa1606468 28002688

[4] Hauser SL, Kappos L, Arnold DL, Bar-Or A, Brochet B, Naismith RT (2020). Five years of ocrelizumab in relapsing multiple sclerosis: OPERA studies open-label extension. Neurology..

[5] Wolinsky JS, Arnold DL, Brochet B, Hartung H-P, Montalban X, Naismith RT, et al. Long-term follow-up from the ORATORIO trial of ocrelizumab for primary progressive multiple sclerosis: a post-hoc analysis from the ongoing open-label extension of the randomised, placebo-controlled, phase 3 trial. Lancet Neurol [Internet]. 2020;1–12. Available from: 10.1016/S1474-4422(20)30342-2 10.1016/S1474-4422(20)30342-2 33129442

[6] Turner B, Cree BAC, Kappos L, Montalban X, Papeix C, Wolinsky JS, et al. Ocrelizumab efficacy in subgroups of patients with relapsing multiple sclerosis. J Neurol [Internet]. 2019 May 28;266(5):1182–93. Available from: 10.1007/s00415-019-09248-6 10.1007/s00415-019-09248-6 PMC6469695 30820738

[7] Butzkueven H, Spelman T, Horakova D, Hughes S, Solaro C, Izquierdo G, et al. Risk of requiring a wheelchair in primary progressive multiple sclerosis: Data from the ORATORIO trial and the MSBase registry. Eur J Neurol [Internet]. 2021 May 6;ene.14824. Available from: 10.1111/ene.14824 10.1111/ene.14824 PMC9292576 33724638

[8] Giovannoni G, Kappos L, Seze J, Hauser SL, Overell J, Koendgen H, et al. Risk of requiring a walking aid after 6.5 years of ocrelizumab treatment in patients with relapsing multiple sclerosis: Data from the OPERA I and OPERA II trials. Eur J Neurol [Internet]. 2021 May 5;ene.14823. Available from: 10.1111/ene.14823 10.1111/ene.14823 PMC9290576 33724637

[9] Wolinsky JS, Engmann NJ, Pei J, Pradhan A, Markowitz C, Fox EJ (2020). An exploratory analysis of the efficacy of ocrelizumab in patients with multiple sclerosis with increased disability. Mult Scler J - Exp Transl Clin..

[10] Prockl V, Nickel FT, Utz KS, Fröhlich K, Engelhorn T, Hilz MJ, et al. Real world application of ocrelizumab in multiple sclerosis: Single-center experience of 128 patients. J Neurol Sci [Internet]. 2020;415(June):116973. Available from: 10.1016/j.jns.2020.116973 10.1016/j.jns.2020.116973 32563101

[11] Fernandez-Diaz E, Perez-Vicente JA, Villaverde-Gonzalez R, Berenguer-Ruiz L, Candeliere Merlicco A, Martinez-Navarro ML, et al. Real-world experience of ocrelizumab in multiple sclerosis in a Spanish population. Ann Clin Transl Neurol. 2020;1–10. 10.1002/acn3.51282 PMC7886031 33369288

[12] Sempere AP, Berenguer-Ruiz L, Borrego-Soriano I, Burgos-San Jose A, Concepcion-Aramendia L, Volar L, et al. Ocrelizumab in Multiple Sclerosis: A Real-World Study From Spain. Front Neurol [Internet]. 2021 Jan 15;11. Available from: 10.3389/fneur.2020.592304/full 10.3389/fneur.2020.592304 PMC7844090 33519676

[13] Patel A, Sul J, Gordon ML, Steinklein J, Sanguinetti S, Pramanik B, et al. Progressive Multifocal Leukoencephalopathy in a Patient With Progressive Multiple Sclerosis Treated With Ocrelizumab Monotherapy. JAMA Neurol [Internet]. 2021 Mar 16; Available from: https://jamanetwork.com/journals/jamaneurology/fullarticle/2777642 10.1001/jamaneurol.2021.0627 PMC7967248 33724354

[14] Machlańska A, Helbig G, Chromik K, Zapała M, Zwiernik B, Selmaj K. Hemophagocytic lymphohistiocytosis associated with ocrelizumab treatment in a patient with multiple sclerosis. Mult Scler J [Internet]. 2021 Mar 5;135245852199307. Available from: 10.1177/1352458521993070 10.1177/1352458521993070 33666121

[15] Brownlee W, Bourdette D, Broadley S, Killestein J, Ciccarelli O. Treating multiple sclerosis and neuromyelitis optica spectrum disorder during the COVID-19 pandemic. Neurology [Internet]. 2020 Jun 2;94(22):949–52. Available from: 10.1212/WNL.0000000000009507 10.1212/WNL.0000000000009507 32241953

[16] MS International Federation. Global COVID-19 advice for people with MS. 2020.

[17] Rush CA, MacLean HJ, Freedman MS. Aggressive multiple sclerosis: proposed definition and treatment algorithm. Nat Rev Neurol [Internet]. 2015 Jul 2;11(7):379–89. Available from: 10.1038/nrneurol.2015.85 10.1038/nrneurol.2015.85 26032396

[18] Barmettler S, Ong M-S, Farmer JR, Choi H, Walter J. Association of Immunoglobulin Levels, Infectious Risk, and Mortality With Rituximab and Hypogammaglobulinemia. JAMA Netw Open [Internet]. 2018 Nov 2;1(7):e184169. Available from: 10.1001/jamanetworkopen.2018.4169 10.1001/jamanetworkopen.2018.4169 PMC6324375 30646343

[19] He A, Merkel B, Brown JWL, Zhovits Ryerson L, Kister I, Malpas CB, et al. Timing of high-efficacy therapy for multiple sclerosis: a retrospective observational cohort study. Lancet Neurol [Internet]. 2020 Apr;19(4):307–16. Available from: https://linkinghub.elsevier.com/retrieve/pii/S1474442220300673 10.1016/S1474-4422(20)30067-3 32199096

[20] Buron MD, Chalmer TA, Sellebjerg F, Barzinji I, Christensen JR, Christensen MK, et al. Initial high-efficacy disease-modifying therapy in multiple sclerosis. Neurology [Internet]. 2020 Aug 25;95(8):e1041–51. Available from: 10.1212/WNL.0000000000010135 10.1212/WNL.0000000000010135 32636328

[21] Brown JWL, Coles A, Horakova D, Havrdova E, Izquierdo G, Prat A, et al. Association of Initial Disease-Modifying Therapy With Later Conversion to Secondary Progressive Multiple Sclerosis. JAMA [Internet]. 2019 Jan 15;321(2):175. Available from: https://jamanetwork.com/journals/jama/fullarticle/2720726 10.1001/jama.2018.20588 PMC6439772 30644981

[22] Barkhof F, Kappos L, Wolinsky JS, Li DKB, Bar-Or A, Hartung HP (2019). Onset of clinical and MRI efficacy of ocrelizumab in relapsing multiple sclerosis. Neurology..

[23] Havrdová E, Arnold DL, Bar-Or A, Comi G, Hartung H-P, Kappos L, et al. No evidence of disease activity (NEDA) analysis by epochs in patients with relapsing multiple sclerosis treated with ocrelizumab vs interferon beta-1a. Mult Scler J - Exp Transl Clin [Internet]. 2018 Jan 12;4(1):205521731876064. Available from: 10.1177/2055217318760642 10.1177/2055217318760642 PMC5858626 29568544

[24] Signoriello E, Lus G, Bonavita S, Lanzillo R, Saccà F, Landi D, et al. Switch from sequestering to anti-CD20 depleting treatment: disease activity outcomes during wash-out and in the first 6 months of ocrelizumab therapy. Mult Scler J [Internet]. 2021 Apr 15;135245852110056. Available from: 10.1177/13524585211005657 10.1177/13524585211005657 33855897

[25] Schuh E, Berer K, Mulazzani M, Feil K, Meinl I, Lahm H, et al. Features of Human CD3 + CD20 + T Cells. J Immunol [Internet]. 2016 Aug 15;197(4):1111–7. Available from: 10.4049/jimmunol.1600089 10.4049/jimmunol.1600089 27412413

[26] Gingele S, Skripuletz T, Jacobs R. Role of CD20 + T cells in multiple sclerosis: implications for treatment with ocrelizumab. Neural Regen Res [Internet]. 2020;15(4):663. Available from: http://www.nrronline.org/text.asp?2020/15/4/663/266913 10.4103/1673-5374.266913 PMC6975146 31638088

[27] Gingele S, Jacobus T, Konen F, Hümmert M, Sühs K-W, Schwenkenbecher P (2018). Ocrelizumab Depletes CD20+ T Cells in Multiple Sclerosis Patients. Cells..

[28] Fernández-Velasco JI, Kuhle J, Monreal E, Meca-Lallana V, Meca-Lallana J, Izquierdo G, et al. Effect of Ocrelizumab in Blood Leukocytes of Patients With Primary Progressive MS. Neurol - Neuroimmunol Neuroinflammation [Internet]. 2021 Mar 4;8(2):e940. Available from: 10.1212/NXI.0000000000000940 10.1212/NXI.0000000000000940 PMC7862094 33408167

[29] Palanichamy A, Jahn S, Nickles D, Derstine M, Abounasr A, Hauser SL, et al. Rituximab Efficiently Depletes Increased CD20-Expressing T Cells in Multiple Sclerosis Patients. J Immunol [Internet]. 2014 Jul 15;193(2):580–6. Available from: 10.4049/jimmunol.1400118 10.4049/jimmunol.1400118 PMC4082756 24928997

[30] Capasso N, Nozzolillo A, Scalia G, Lanzillo R, Carotenuto A, De Angelis M, et al. Ocrelizumab depletes T-lymphocytes more than rituximab in multiple sclerosis. Mult Scler Relat Disord [Internet]. 2021 Apr;49:102802. Available from: https://linkinghub.elsevier.com/retrieve/pii/S2211034821000687 10.1016/j.msard.2021.102802 33556652

[31] Seery N, Sharmin S, Li V, Nguyen A-L, Meaton C, Atvars R, et al. Predicting Infection Risk in Multiple Sclerosis Patients Treated with Ocrelizumab: A Retrospective Cohort Study. CNS Drugs [Internet]. 2021 Apr 13; Available from: 10.1007/s40263-021-00810-3 10.1007/s40263-021-00810-3 PMC8042832 33847902

[32] Hills T, Kearns N, Kearns C, Beasley R. Influenza control during the COVID-19 pandemic. Lancet [Internet]. 2020 Nov;396(10263):1633–4. Available from: https://linkinghub.elsevier.com/retrieve/pii/S0140673620321668 10.1016/S0140-6736(20)32166-8 PMC7581384 33228919

